# Introduction of a Stick or Swab-handle More Than Ten Inches Long into the Stomach—Its Exit by an Abscess—Cure

**Published:** 1855-02

**Authors:** Francisco Garcia Y Garcia

**Affiliations:** Daimiel (Mancha baja)


*Introduction of a stick or swab-handle more than ten inches long into the stomach—its exit by an abscess—cure.* By FRANCISCO GARCIÀ Y GARCIA, of Daimiel (Mancha baja.)

(Translated for the Medical Examiner from El Porvenir Medico of June, 1854.)

Mateo Sanchez de la Nieta, native of the town of Daimiel' aged between 45 and 50 years, contracted a syphilitic disease' which, after a time, affected the fauces and posterior part of the mouth. His attending physician, a distinguished practitioner, directed that the parts should be cleansed several times daily, and for the purpose constructed a swab with which he made the first applications, but not being sufficiently long the patient had it spliced until the stick was more than ten inches (una tercia) in length.

One afternoon in the month of September, he was alone in his house and complying with the directions of the practitioner; but the presence of the swab, the stimulus of the medicament, the contraction of the muscles of the pharynx, a spasmodic movement, the carelessnesss of the patient, or all conjoined, caused him to relinquish his grasp of the instrument, which remained in the back part of the mouth. While thus embarrassed, one of his daughters came in, who perceiving him in distress, and not able to answer questions, gave him water, which he asked for by signs, which not being able to swallow, was returned by the mouth and nostrils, with suffocating effect; some persons in the vicinity seeing him sought a physician; in the mean time, which was not long, the stick descended the œsophagus, the upper extremity fixing itself between the pomum Adami and the anterior portion of the fork formed by the sterno-cleido mastoideus, producing a salient angle on the left side, which the patient indicated to the bystanders. A suffocating condition recurring, caused him to abandon it, and with the movements it disappeared not only from sight but also from the touch of the practitioner, who arrived after the patient had recovered from the paroxysm which followed that state. They gave him some spoonfuls of an anti-spasmodic mixture, which he swallowed with less difficulty than the water given him by his daughter. He recovered his speech somewhat, and complained only of anguish and smarting in the throat, and towards the left side a little above and front of the nipple of the same side, which gradually ceased, disappearing on the fourth day, the patient, physician and friends being left in an unhoped for quiet.

Eight days subsequently the patient felt, deep in the left side of the epigastric region, sharp pains, running towards the last false ribs, increasing every hour, accompanied with gastric irritation and febrile symptoms, which led the physician to suggest a resort to spiritual aid. On the following day, (the 14th from the ingestion of the stick) the greater part of the gastro-peritoneal symptoms, which indicated great peril, abated, the patient remaining almost without fever from the 17th to the 20th. Under these circumstances the practitioner proceeded to a minute examination of the patient, and ascertaining the existence of the stick in the cavity of the stomach, proposed to the patient the operation of *gastrotomy*, to which he objected his age, his severe suffering, his present comfortable condition, and finally that he would not submit, though it would cost him his life.

The practitioner forced unwillingly to yield to the entreaties of the patient, and abandon all operations, directed him to eat, assuring him his condition was not as flattering as he supposed. At the expiration of ten days (26th of the accident) the patient presented himself at the house of the physician, asking him to examine an *apostume,* as he called it, which had appeared far below the nipple of the left side. The next day it was opened by a crucial incision, and a large quantity of pus, both well formed and bloody, was discharged; with the evacuation the patient grew worse, but four days after the incision, having improved somewhat, and feeling himself much better, without waiting for the physician, he determined to remove the dressing and cleanse the wound: a female neighbor, who was present to assist him, saw with wonder what appeared to be the end of a black stick in the opening of the abscess; encouraged by the patient, she seized the foreign body and drew it, and they saw with astonishment four or five inches of the stick of the swab projecting.

In the midst of the conflict of the two, they thought of and sent for Dr. Povil, who came at once to the aid of his patient; he took the stick,—and assisted by the exit of pus, contractile movements of the stomach, muscles of inspiration and traction of the woman,—brought it to the surface, in *the intercostal space formed between the third and fourth false ribs of the left side*, as far as the point where it was spliced; then he seized the stick at the splice, fearing that the thread which bound it might give way, in consequence of putrefaction, which he presumed might have occurred since its ingestion: but this fear vanished when the point of the splice passing through the intercostal space, the thread was found unaltered; the extraction was continued until the extremity of the stick, to which threads or a frayed rag were tied, reached the external wound, where it stuck, causing new and sharp pains in the stomach, which, although they subsided, were followed by great distress and a soporose state. Having recovered, the practitioner continued his exertions, and introduced his thumb into the wound, and by forcibly depressing the inferior rib, succeeded in dislodging and extracting the entire swab just as it had entered the mouth 28 days before. It was followed by a flow of pus, considerable blood, and gastric juice through the wound, together with some partially digested alimentary substances which had been eaten in the morning.

Care was taken in dressing the wound to avoid the introduction of air into it. He was placed upon his back with head and shoulders elevated, ordered a strict diet, being allowed a few spoonsful of acidulated water for drink. The stick was found to be of black poplar, (Populus nigra), and more than a tercia (a third of a Spanish yard) or about eleven inches in length. The patient passed an uncomfortable night, but slept at intervals in the early part of the next morning. On the 4th day from the removal of the stick the wound was of a dark color, owing to the presence of some coagula of blood ; these came away the next day with the poultice, and the wound assumed a healthy appearance, and was completely healed in 26 days from the removal of the stick, and 49 from its entrance by the mouth.

This case occurred at Daimiel in the month of September, 1832, and was well known among the people. The statement is from the patient before death and from his children who witnessed it. In 1834 Sanchez had a light attack of cholera; but during the ten succeeding years he worked as gardener and laborer without suffering from any serious indisposition; he remained fat and healthy until May 1844, when the writer began to practice in the town. He died in 1849 of an acute attack of pleuro-pneumonia. [W. S. W. R.]

